# Relationship between Physical Activity, Screen Time and Weight Status among Young Adolescents

**DOI:** 10.3390/sports6030057

**Published:** 2018-06-23

**Authors:** Wesley O’Brien, Johann Issartel, Sarahjane Belton

**Affiliations:** 1 School of Education, Sports Studies and Physical Education Department, 2 Lucan Place, Western Road, University College Cork, Cork T12 KX72, Ireland; 2 Centre of Preventive Medicine, School of Health and Human Performance, Dublin City University, Dublin D09 W6Y4, Ireland; johann.issartel@dcu.ie (J.I.); sarahjane.belton@dcu.ie (S.B.)

**Keywords:** overweight, obese, sedentary behaviour, accelerometer

## Abstract

It is well established that lack of physical activity and high bouts of sedentary behaviour are now associated with all-cause and cardiovascular mortality. The purpose of this study was to investigate the relationship between physical activity participation, overall screen time and weight status amongst early Irish adolescent youth. Participants were a sample of 169 students: 113 boys (mean age = 12.89 ± 0.34 years) and 56 girls (mean age = 12.87 ± 0.61 years). The data gathered in the present study included physical activity (accelerometry), screen time (self-report) and anthropometric measurements. Overweight and obese participants accumulated significantly more minutes of overall screen time daily compared to their normal-weight counterparts. A correlation between physical activity and daily television viewing was evident among girls. No significant interaction was apparent when examining daily physical activity and overall screen time in the prediction of early adolescents’ body mass index. Results suggest the importance of reducing screen time in the contribution towards a healthier weight status among adolescents. Furthermore, physical activity appears largely unrelated to overall screen time in predicting adolescent weight status, suggesting that these variables may be independent markers of health in youth. The existing relationship for girls between moderate-to-vigorous physical activity and time spent television viewing may be a potential area to consider for future intervention design with adolescent youth.

## 1. Introduction

Physical activity (PA) is now perceived as the cornerstone in the physical development of infants, young and older children, and adolescents approaching adulthood [1]. Interestingly, the meaning of PA has remained consistent amongst public health professionals over the last three decades, and a standardised PA definition has become accepted as any bodily movement produced by the skeletal muscles that result in a substantial increase over resting energy expenditure [2]. Recent evidence [3] highlights that an increase in PA levels equivalent to brisk walking for a minimum of 75 min per week is associated with a gain of 1.8 years in life expectancy. Contrary to PA, ‘‘sedentary living’’ reduces longevity [4] and is associated with significant health risks. In recent years, sedentary pursuits such as television viewing, playing computer games and using the internet have become the preferred mode of passive entertainment in daily living among youth [5,6]. Surveillance data from a European perspective indicate that youth’s PA levels have substantially decreased [7] with the proportion of obese children having dramatically increased in the last 30 years [8]. 

As reported by Belton et al. [9], the most widely endorsed PA guideline stipulates that in order to enhance health, adolescent youth should accumulate at least 60 min of moderate-to-vigorous PA (≥60 min MVPA) daily [10]. There are now numerous studies to suggest that levels of PA are falling short of recommended minimum guidelines and that levels decline during adolescence [7,11,12]. A previous Irish study on the activity levels of young people indicates that only 12% of adolescent youth meet the PA guidelines [13]. With these documented low levels of youth PA engagement, research now highlights relationships between low levels of PA, increased levels of sedentary pursuits and body mass index (BMI) among children and adolescents [14]. 

The concept of sedentary behaviour is not a new phenomenon [5]; findings from the past decade indicate that higher levels of sedentary behaviours are negatively associated with cardiometabolic risk factors [15,16]. Previously, sedentary behaviour referred to activities that did not increase energy expenditure substantially above the resting level and included activities such as sleeping, sitting, lying down and watching television [17]. Most recently, however, sedentary behaviour has been defined as any waking behaviour (independent of sleep) that is characterized by an energy expenditure ≤1.5 metabolic equivalents (METs) while in a sitting, reclining or lying posture [18]. The thematic field of sedentary behaviour has gathered momentum in recent years [19], with studies addressing sedentary behaviours amongst youth due to the attraction of electronic games, computer use and the over-reliance of screen-based activity engagement [5]. A recent study found that a reduction in television viewing during adolescence, in addition to regular PA, may improve cardiometabolic health (heart and metabolic functioning) later in life [20]. While the independent effects of sedentary behaviour on health outcomes can vary in degree of magnitude [21], this concept is of considerable importance, warranting further study and examination in young people [18]. 

There is a need to develop specified sedentary behaviour guidelines for children and youth in Ireland and worldwide [13]. The Canadian Sedentary Behaviour Guidelines for Children (5–11 years of age) and Youth (12–17 years of age) previously published recommendations stating that for health benefits, recreational screen time [18] should be limited to no more than 2 h per day. The Health Behaviour in School-aged Children (HBSC) report [7] found that between 63% and 65% of adolescents (13 to 15 year olds) worldwide watch television for more than 2 h per day on weekdays. From an Irish perspective, this concerning trend towards sedentary behaviour seems most apparent with less than 1% of children and youth meeting the health recommendations of no more than 2 h of screen time during daylight hours [13]. 

The present study seeks to examine possible differences in both PA participation and screen time behaviours according to the weight status of early Irish adolescent youth. Separate analyses have been conducted for boys and girls as previous evidence amongst young people suggests clear gender differences in PA, screen time and weight status [22,23]. Considering that previous cross-sectional research has found associations between lower levels of PA participation, greater periods of screen time engagement and higher levels of BMI amongst youth [14,24], investigating the relationship between these variables is a timely issue that could inform obesity prevention strategies for adolescents [25]. This present investigation serves as a comparison for existing studies and, to our knowledge, has never been reported previously amongst an Irish adolescent population.

## 2. Methods

### 2.1. Study Design and Participants

This Irish cross-sectional study is part of a larger longitudinal study entitled the Youth-Physical Activity Towards Health (Y-PATH) research programme [9,26,27,28,29], which was initiated in September 2010. Baseline data for the present study were gathered in September 2011, which specifically included PA (accelerometry), screen time (self-report) and basic anthropometric measurements (height and weight).

A convenience sample of 169 Irish adolescents (n = 113 boys, mean age = 12.89 ± 0.34 years; n = 56 girls, mean age = 12.87 ± 0.61 years) enrolled in year one of post-primary education, from two mixed gender rural schools, participated in the present study. Regarding school-level information, both mixed gender rural schools were of typical socioeconomic status in the Republic of Ireland. There were no socioeconomic status differences between both school levels and those included for participation in the current study were not from the Republic of Ireland Delivering Equality of Opportunity in Schools (DEIS) index. Approval from the principals of the two participating schools was granted. Informed consent for participation was sought from all adolescents and their parents/guardians. Ethical approval was obtained from the University Research Ethics Committee (DCUREC/2010/081).

Twenty of the original 169 participants were subsequently omitted from the BMI datasets, specifically as a result of missing data. BMI was collected on a separate day to the questionnaire and accelerometer measurements in both schools due to timetable restrictions. Of the original 169 participants who completed the questionnaire and wore accelerometers, 11.8% (n = 20) were unavailable or not present on the second day in the school setting for BMI data collection. Furthermore, due to the stringent accelerometer inclusion protocol, as reported previously for habitual PA measurement [27], 74 participants (43.8%) did not meet the minimum wear time criteria (reported below) for adolescent youth. 

### 2.2. Measurement

Moderate-to-vigorous physical activity: Moderate-to-vigorous physical activity (MVPA) was objectively measured for one hundred and sixty-nine (169) participants during nine consecutive days using two types of accelerometers (GT1M and GT3X models - Actigraph, FL, United States); previous research has reported that it is reasonable to compare data derived from both models of accelerometer when collected in the uniaxial mode [30]. Participants were shown how to wear the accelerometer above the iliac crest of the right hip, as recent evidence suggests the highest percentage of correctly classified activities is achieved when using data from the hip [31]. Participants were asked to wear the accelerometer during all waking hours unless showering, swimming or taking part in a contact activity for which an adult deemed it unsafe to wear. 

As part of this study, using the protocol from Belton et al., [32] an investigator checked in at the school each morning between 9:00 a.m. and 10:00 a.m. to ensure participants were wearing the accelerometer monitors correctly. In the event that a child had forgotten to wear his/her device, their parents/guardians were contacted to drop off the device within the first 2 h of school. The compliance strategy employed was to send a ‘short message service’ (SMS) reminder message before 8 a.m. each weekday morning (9:30 a.m. on weekend days) and after 4 p.m. each weekday afternoon (5 p.m. on weekend days). Due to ethical reasons, students self-selected whether to provide their mobile number or their parent/guardian’s mobile number to receive the reminder SMS strategy.

In line with previous recommendations [33], the first and last day of accelerometer data were omitted from analysis. PA was recorded in 10-s epochs to capture the intermittent and sporadic behaviour of youth [34]. Minutes in moderate and vigorous PA were estimated from the data using the validated Evenson cut-points for the youth of this age group [35]: moderate PA ≥2296 and vigorous PA ≥4012 (all values are counts per minute). Monitor non-wear periods were defined as ≥20 consecutive minutes of zero counts [36]. In line with other studies, a day was deemed valid (and thereby included in analysis) if there was a minimum of 600 min recorded wear time per day [37]. The minimum number of valid days required for inclusion in the analysis was 3 weekdays and 1 weekend day [38]. 

Screen Time: Screen time activities were derived using the Youth Physical Activity Questionnaire (YPAQ) [39], which has been previously validated against accelerometers (0.42) amongst 12- to 13-year-olds. The three types of screen time activities analysed in the present study included (1) television viewing, (2) playing video games and (3) using the computer. Participants were requested to self-report the frequency and duration of each screen time activity for both week and weekend days over the previous 7 days [39]. Overall screen time [40] was calculated by summing the average number of minutes per day for each of the three variables. Data was collected on participants in their class groups (maximum n = 30) during a 2-h school visit, with a ratio of 1 researcher to 15 students for questionnaire completion. In cases where computer networks failed, participants completed hard copies of the questionnaire. A 48-h time sampling test re-test reliability among a sample of 35 participants (12–13 years of age) was carried out to ensure comparability of the two administration protocols (computer versus hardcopy) [9]; reliability coefficients reached 0.94, showing the results across both formats of the questionnaires to be consistent over time. 

Body mass index (BMI): Weight was measured to the nearest 0.1 kg using the Seca 761 dual platform weighing scales, while height was measured to the nearest 0.1 cm using a portable stadiometer (Seca 213, Hanover, MD, United States). The cut-off points defined by the International Obesity Task Force [41] for normal, overweight and obese participants were applied to the data in order to categorise weight status. All of the BMI data were collected by field staff who received one full day of training prior to the data collection rollout. Adhering to ethical gender protocol for height and weight measurements in mixed-gender post-primary schools, two members of field staff (one male, one female) were trained by the principal investigator. The trained field staff were required to reach a minimum of 95% interobserver agreement for each height and weight measurement on a selected sample of participants prior to data collection.

### 2.3. Data Analysis

Data were analysed using SPSS version 17.0 (Chicago, IL, United States) for Windows. All data were checked for normality before statistical analysis. Descriptive statistics and frequencies for the demographic, physical characteristics, PA, types of screen time activities and overall screen time were calculated; when broken down by gender and weight status, descriptive statistics further explored the percentage of participants accumulating at least 30 min, 45 min and 60 min of MVPA daily (specifically based on the average times across valid days of accelerometer data). 

Gender differences in PA, types of screen time activities and overall screen time were analysed using independent sample *t*-tests. Chi-square tests for independence were further used to examine whether gender and weight status differences in PA and screen time recommendations existed. Pearson correlation coefficients examined the strength of the relationship among types of screen time activities and PA, overall screen time and PA, types of screen time activities and BMI scores, overall screen time and BMI scores and PA and BMI scores. Standard multiple regression was performed to examine the overall relationship between the measurement of gender, minute-by-minute activity counts of MVPA and overall screen time in the prediction of adolescent levels of BMI (the included sample for this aspect of the regression analysis, therefore, comprised 84 participants only, providing fully available and inclusive MVPA, overall screen time and BMI data). Statistical significance was set at *p* < 0.05.

## 3. Results

Prior to analysing the findings and to ensure that the data from the two mixed-gender rural schools were representative, independent sample *t*-tests confirmed that there were no significant school-type differences in MVPA, screen time activities and BMI. 

The descriptive data of the physical characteristics according to gender and the type of screen time activities of the sample are presented in Table 1. The mean age of the participants was 12.88 ± 0.45 years with 25.5% (n = 25) of boys and 29.4% (n = 15) of girls classified as overweight and/or obese. There were no significant gender differences between the percentages of overweight or obese individuals (χ^2^ = 0.099, *p* = 0.753, φ = 0.042). In relation to the type of screen time activities, independent sample *t*-tests confirmed that there was a significant gender difference with boys accumulating more minutes (M = 23.32, SD = 53.88) of daily video game usage compared to girls (M = 7.19, SD = 14.34; t (167) = 2.199, *p* = 0.029).

The overall mean daily screen time for participants was 82.24 ± 109.41 min per day (min/d). Table 2 outlines the overall daily screen time and adherence to the associated screen time recommendations according to gender and weight status. Male participants (n = 113) accumulated more minutes (90.76 min/d) of daily screen time than female participants (n = 56; 65.05 min/d); yet, statistically, there were no significant gender differences. When broken down by weight status, overweight/obese participants (n = 40) accumulated 103.5 min/d screen time compared to 64.09 min/d screen time for normal-weight participants (n = 109). Independent *t*-tests subsequently confirmed that there was a significant weight status difference with overweight and obese participants accumulating more minutes per day of screen time than normal-weight participants (t (147) = −2.105, *p* = 0.037).

Descriptive statistics showed that overall 25.3% (n = 24) of participants met the 60 min/d MVPA guideline. The mean min/d MVPA was higher for male participants (n = 59; 53.99 ± 20.00 min/d) than females (n = 36; 39.64 ± 12.78 min/d) with an independent sample *t*-test confirming that boys accumulated significantly more min/d MVPA (t(93) = 3.845, *p* < 0.001). 

**Figure 1:**
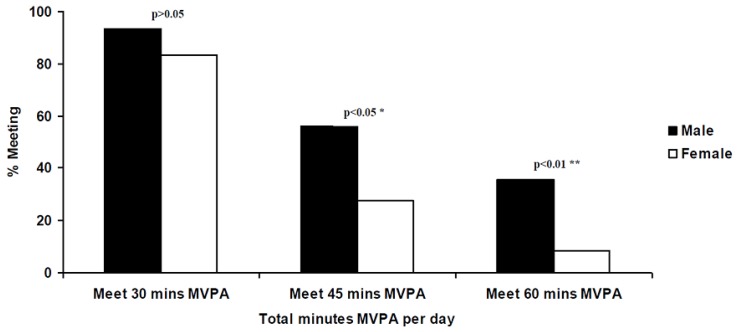
Percentage of male and female participants meeting 30 to 60 min MVPA per day.

**Figure 2:**
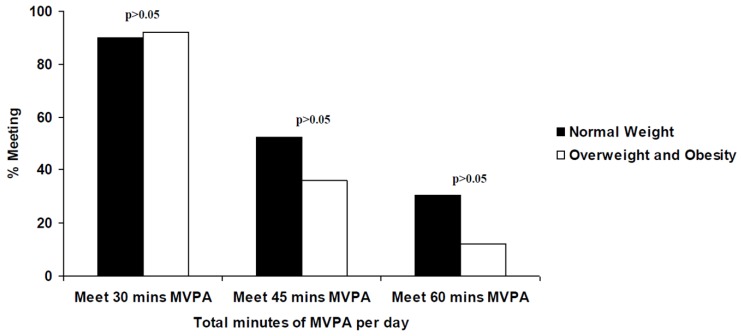
Percentage of normal weight and overweight/obese participants meeting the 30 to 60 min MVPA per day.

Figure 1 and Figure 2 illustrate the prevalence of those accumulating at least 30 min/d, 45 min/d and 60 min/d of MVPA for both gender and weight status. Chi-square tests for independence indicated that significant gender differences were again observed in those who accumulated at least 45 min/d (χ^2^ = 6.062, *p* = 0.014) and 60 min/d MVPA (χ^2^ = 7.415, *p* = 0.006); a statistically higher proportion of boys accumulated at least 45 and 60 min/d MVPA. 

When broken down by weight status, the mean min/d MVPA was higher for normal-weight participants (50.53 ± 18.10 min/d) than those in the overweight and obese categories (45.41 ± 19.22 min/d). Yet, statistically, chi-square tests for independence indicated that weight status had no significant effect on those accumulating at least 30 min/d, 45 min/d and 60 min/d of MVPA. 

Table 3 shows the correlations among types of screen time activities, overall screen time, PA and BMI by gender. Male BMI scores showed a weak but significant positive correlation with playing computer games only (r = 0.20, *p* < 0.05). Among girls, time spent in MVPA showed a significant medium negative correlation with daily television viewing only (r = −0.35, *p* < 0.05).

Finally, standard multiple regression was used to assess the ability of gender, PA and overall screen time to predict adolescent levels of BMI. After the entry of these variables, the model as a whole revealed that gender, PA and overall screen time explained 3% of the variance in the prediction of BMI (F(3, 80) = 0.768, *p* > 0.05), indicating that no significant relationship was apparent.

## 4. Discussion

This article examined whether possible differences in gender, objectively measured PA participation and self-reported screen time varied according to weight status of 12- to 14-year-olds. It further investigated whether an overall interaction effect among daily MVPA, screen time and BMI was emergent among early adolescent youth. 

While the observed levels of PA participation according to weight status were low in general, consistent with recent gender differences in adolescent PA [12,13] and in movement-based literature [42], the present findings indicate that adolescent boys accumulate significantly more minutes of MVPA than girls and are significantly more likely to meet the recommended PA guidelines for health. In contrast to these male PA results, this study found that adolescent girls accumulated significantly fewer minutes of daily video game usage than boys did. The finding of the present study compares to a similar study examining the relationship between sedentary activities and physical inactivity among adolescents [23] in which girls accumulated substantially less video game time than boys. This may in part be explained by the fact that traditionally video games have been perceived to belong in the male domain, which lends support to the solution of replacing screen time with desirable recreational activities [43] for increased PA participation among girls. 

While the prevalence of overweight and obese participants (25.5% male; 29.4% female) in the current study are in line with the nationally representative data for an adolescent population in Ireland (25%) [13], it was further interesting to observe in the current study that an increase in weight status may have an association on the accumulated minutes of daily screen time engagement (see Table 2). Previous hypotheses [44,45] suggested that screen time behaviour, such as television viewing, may be a contributing factor among a constellation of pathways to obesity because it may displace PA, increase calorie consumption and reduce resting metabolism. The present result implies that interventions aimed at reducing screen time may be helpful in further understanding body composition amongst youth. As defined by Tremblay et al., [18], ‘active screen time’ refers to the time spent on screen-based behaviours and how these behaviours can be performed while being either sedentary or physically active. For these reasons, the effectiveness of active screen time on adolescent body composition, in comparison to sedentary screen time, warrants further attention in the literature. 

Due to the cross-sectional design of this study, a cause-and-effect relationship between PA and screen time cannot be determined. For this reason, interpreting the result that highlighted a significant negative relationship between female MVPA and daily television viewing (see Table 3) has proven difficult. Recent evidence [46] suggests that screen time viewing and PA have no association in elementary school children aged between 6 and 11 years; nonetheless, both variables were found to be independently associated with obesity status. The present result for female adolescents is consistent with a previous study [44] in which it was shown that after-school television viewing was weakly negatively associated (r = −0.086, *p* = 0.026) with PA levels in adolescent girls. While the association found in the present study was slightly stronger, the result is difficult to interpret from a female perspective only. This finding between MVPA and daily television viewing for girls cannot be dispelled; further research is warranted in order to counter the compelling evidence reported in previous studies [46] that PA and sedentary behaviour are unrelated [22] and subsequently do not displace one another [19]. 

Finally, no overall interaction effect was found between daily MVPA participation and screen time in the prediction of early adolescent BMI in this study. This finding contradicts a recent study among 10- to 12-year-old boys and girls in Europe [22], whereby it was found that higher bouts of MVPA and less engagement time within sedentary behaviour were associated with a healthier weight status. Findings of the current study can be compared to a previous study [25], who also found no significant association when assessing the relationship between 2-year changes in objectively measured PA, sedentary behaviour, and BMI in a younger cohort of 7- to 9-year-old children. Equally, previous other cross-sectional and longitudinal studies [47] have found non-significant associations between PA, sedentary behaviour and BMI. Findings lend support to the argument that adolescent sedentary behaviour and PA are unrelated and may not be two sides of the same coin [22]. 

Limitations of the current study include the cross-sectional research design of two rural Irish post-primary schools, the lack of adjustment for covariates (such as puberty status), and the low number of overweight, obese, and female participants (sample skewed to 67% male) with available data. In addition, the relatively low proportion (due to noncompliance) of participants with accelerometer datasets meeting the inclusion criteria can also be considered a weakness within the interpretation of the objective PA. Furthermore, while simple non-invasive height and weight anthropometric measures were undertaken by trained field staff to determine adolescent weight status, the use of BMI as an indicator of weight status may also be considered a limitation. Had an alternative measure such as a fat mass index been used, researchers may have been able to derive additional information regarding body compartmental fat mass in adolescents (Steele et al., 2009). 

A major strength of this study is the rigorous assessment of adolescent PA behaviour via accelerometry, and the use of stringent and widely endorsed inclusion criteria [33,37,38]. To gather information on sedentary behaviour, the authors investigated multiple screen time behaviours as a combination marker for health risk. A previous study [48] highlighted how examining television viewing only appears to be an unrepresentative marker of sedentary behaviour in adolescents. Thus, the present study went beyond the prevalence of television viewing in an attempt to understand this complex behaviour.

## 5. Conclusions

This article extends the debate about the relationship between adolescent PA, screen time and weight status. Findings imply that collectively the variables of MVPA participation and screen time have little or no effect in the prediction of BMI. Results further lend support to previous research that found no association between adolescent sedentary behaviour and PA [22], highlighting that both variables can be regarded as being independent to some extent. More recently, however, Saunders et al. [21] in their systematic review have observed that children and youth with high PA participation and low sedentary behaviour appear to have desirable measures of adiposity and cardiometabolic health. 

Despite these results, recent recommendations suggest that it is prudent to further examine whether sedentariness displaces PA [19]. While causality cannot be inferred from these data, results do suggest the importance of reducing screen time in the contribution towards a healthier weight status among adolescents. The existing relationship for girls found in this study between MVPA and time spent television viewing is of some importance. Consistent with recent findings [6], it seems plausible that girls may benefit most from interventions focusing simultaneously on a decrease in television viewing and the promotion of daily MVPA. Future strategies promoting youth PA should statistically examine the mediating effects of interventions for the most effective implementation of programmes.

## Figures and Tables

**Table 1:** Descriptive data for the physical characteristics and types of screen time activities according to gender.

Variable	Boys (n = 113)		Girls (n = 56)		t	*p*-Value
	M	SD	M	SD		
Age (years)	12.89	0.34	12.87	0.61	0.275	0.784
Min/d television viewing	44.96	52.78	35.20	33.96	1.259	0.210
Min/d video game usage	23.32	53.88	7.19	14.34	2.199	0.029 *
Min/d computer usage	22.47	48.92	22.65	28.31	-0.026	0.980
	**Boys (n = 98)**		**Girls (n = 51)**		**t**	***p*-Value**
	**M**	**SD**	**M**	**SD**		
Height (m)	1.55	0.09	1.55	0.06	0.0311	0.756
Weight (kg)	49.15	11.10	49.48	9.99	−0.177	0.860
BMI (kg/m^2^)	20.22	3.38	20.48	3.15	−0.450	0.653

Note. M = mean; SD = standard deviation; Min/d = minutes per day; BMI = body mass index; * *p* ≤ 0.05.

**Table 2:** Overall screen time and adherence to the associated recommendations according to gender and weight status.

Variable	Boys (n = 113)		Girls (n = 56)		t	*p*-Value
	M	SD	M	SD		
Min/d overall screen time	90.76	126.78	65.05	58.17	1.442	0.151
					**χ^2^**	***p*-Value**
Met screen time recommendations ≤ 2 h/d (%)	75.2%		82.1%		0.670	0.413
	**Normal Weight (n = 109)**		**Overweight/Obese (n = 40)**		**t**	***p*-Value**
	**M**	**SD**	**M**	**SD**		
Min/day overall screen time	64.09	57.37	103.50	171.93	−2.105	0.037 *
					**χ^2^**	***p*-Value**
Met screen time recommendations ≤ 2 h/d (%)	80.7%		77.5%		0.042	0.837

Note. M = mean; SD = standard deviation; Min/d = minutes per day; h/d = hours per day. Gender and weight status differences in daily minutes screen time examined using independent *t*-tests; differences in % who met screen time recommendations examined using chi-square tests for independence; * *p* ≤ 0.05.

**Table 3:** Coefficients for correlation among types of screen time activities, PA and BMI scores according to gender.

Correlation Variables	Daily Television Viewing	Daily Video Games	Daily Computer Usage	Overall Screen Time
**Male**				
Moderate PA *p*/day	0.021	−0.211	0.042	−0.016
Vigorous PA *p*/day	−0.062	−0.227	−0.101	−0.126
MVPA *p*/day	−0.031	−0.211	−0.051	−0.072
BMI scores	−0.077	0.201*	0.018	0.087
**Female**				
Moderate PA *p*/day	−0.260	0.274	−0.004	−0.088
Vigorous PA *p*/day	−0.341	0.259	0.127	−0.084
MVPA *p*/day	−0.348 *	0.330	0.081	−0.087
BMI scores	0.041	−0.168	0.028	0.034

Note. *p*/day = per day; PA = physical activity; MVPA = moderate to vigorous physical activity. * *p* ≤ 0.05.
